# Quo vadis blood protein adductomics?

**DOI:** 10.1007/s00204-021-03165-2

**Published:** 2021-11-13

**Authors:** Gabriele Sabbioni, Billy W. Day

**Affiliations:** 1 Institute of Environmental and Occupational Toxicology, CH-6780 Airolo, Switzerland; 2 Walther-Straub-Institute of Pharmacology and Toxicology, Ludwig-Maximilians-Universität München, 80336 München, Germany; 3 Medantox LLC, Pittsburgh, PA 15241 USA; 4 ReNeuroGen LLC, Elm Grove, WI, 53122 USA

**Keywords:** Biomonitoring, Albumin adducts, Hemoglobin adducts, Adductomics, Carcinogens

## Abstract

Chemicals are measured regularly in air, food, the environment, and the workplace. Biomonitoring of chemicals in biological fluids is a tool to determine the individual exposure. Blood protein adducts of xenobiotics are a marker of both exposure and the biologically effective dose. Urinary metabolites and blood metabolites are short term exposure markers. Stable hemoglobin adducts are exposure markers of up to 120 days. Blood protein adducts are formed with many xenobiotics at different sites of the blood proteins. Newer methods apply the techniques developed in the field of proteomics. Larger adducted peptides with 20 amino acids are used for quantitation. Unfortunately, at present the methods do not reach the limits of detection obtained with the methods looking at single amino acid adducts or at chemically cleaved adducts. Therefore, to progress in the field new approaches are needed.

**Supplementary Information:**

The online version contains supplementary material available at 10.1007/s00204-021-03165-2.

## Introduction

**Fig. 1:**
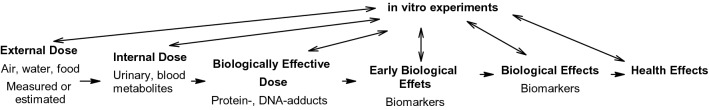
Biomonitoring, biomarkers, and biological effects compared to the results of in vitro experiments

Humans are exposed to xenobiotics through air, water, food, and the environment (Fig. 1). The external dose is determined regularly for a few compounds in air, water, and food by the respective authorities. Computer models have been established to estimate the potential exposure of people (Egeghy et al. 2016). In biomonitoring programs, usually the parent compounds or their metabolites are measured in urine (LaKind et al. 2019). Such measurements are per nature highly variable due to the fast elimination of non-persistent chemicals from the body. The US-EPA (Breen et al. 2021; Dawson et al. 2021; Honda et al. 2019; Wambaugh et al. 2018) and -NIEHS (NTP, https://ice.ntp.niehs.nih.gov/) are working on models to establish a link between in vitro and in vivo data. Using the framework of adverse outcome pathways (AOP), the data obtained in vitro could be used to predict the levels in biological samples (urine, blood) that yield adverse effects in humans (in vitro to in vivo extrapolation (IVIVE)). These predicted levels could be compared to the data obtained in all major existing biomonitoring studies. In the US Centers for Disease Control (CDC)’s National Health and Nutrition Examination Survey (NHANES) studies, health-related parameters have been registered. First studies were performed to link the predicted and effective actual effects obtained mainly from the NHANES program and from medicinal drugs (Honda et al. 2019; Wambaugh et al. 2018). Pharmacological models could be compared. The method could be also applied for the prioritization of chemicals, but more work is needed. However, such evaluations should also be applied to data regarding blood protein and/or DNA adducts.

**Fig. 2:**
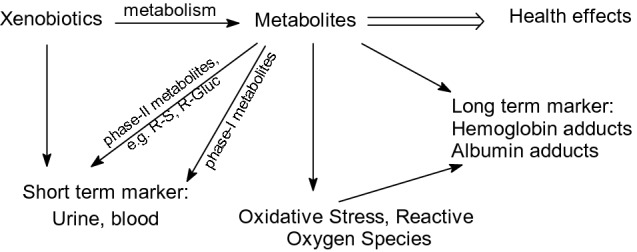
Formation and reaction of reactive metabolites. R = xenobiotics, RS = mercapturic acid, R-Gluc = R-glucuronide

Urinary and blood levels reflect the exposure to non-persistent chemicals of the last 24–48 h. Hair levels of xenobiotics describe the exposure to xenobiotics over a longer time frame. Many chemicals become toxic only after metabolism (Fig. 2). Reactive metabolites form covalent adducts with biomolecules (glutathione, proteins, DNA). This can lead to cytotoxic and genotoxic effects. It is important for the risk assessment of chemicals to quantify the presence of reactive metabolites in the human body. Almost 50 years ago, it was shown that ethylene oxide reacts with hemoglobin and with the DNA of the target organ in a dose-dependent matter (Ehrenberg et al. 1974). Therefore, hemoglobin or albumin adducts of xenobiotics are important dosimeters to monitor the presence of toxic metabolites in the human body (Fig. 2). Stable blood protein adducts reflect the exposure history over a longer time period than do urinary metabolites, or than metabolites present in blood. Stable hemoglobin adducts have a lifetime of up to 120 days and stable albumin adducts a half-life of 20–25 days (reviewed in Sabbioni and Jones 2002; Skipper and Tannenbaum 1990; Törnqvist et al. 2002)) in humans. Reaction products with hemoglobin accumulate up to 60 times a single daily dose and albumin adducts up to 29 times a single daily dose. Blood protein adducts are excellent markers of exposure.

Albumin adduct formation is investigated to determine the potential of drugs for idiosyncratic effects (Baillie 2020; Stepan et al. 2011). Peptide and protein binding tests are included in OECD-tests to evaluate the potential skin sensitization by chemicals (OECD 2021a; OECD 2021b). In the field of occupational and environmental toxicology, binding to proteins is of interest to determine the bioavailability of reactive xenobiotics.

**Fig. 3:**
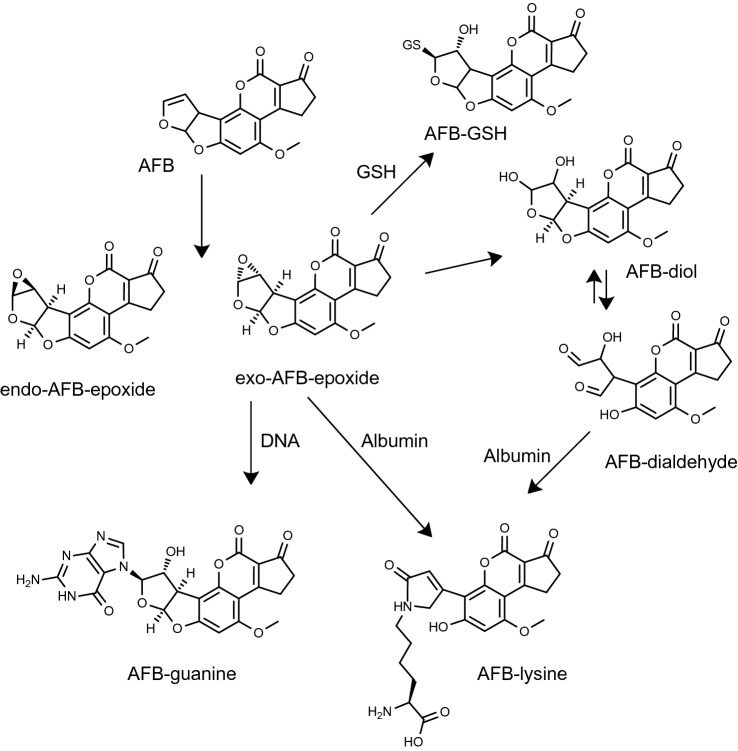
DNA and albumin adducts of aflatoxin B1 (Guengerich et al. 2002; Sabbioni 1990; Sabbioni and Sepai 1998). Reaction of the N6 atom of Lys with the exo-AFB-epoxide is possible but probably does not contribute substantially to the formation of AFB-Lys (Guengerich et al. 2002)

**Fig. 4:**
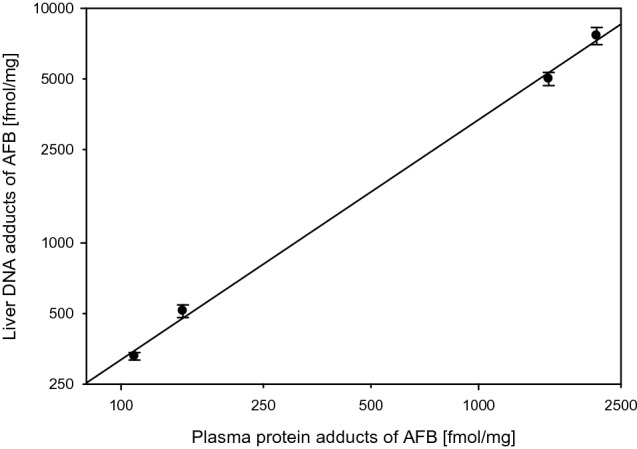
Correlation of DNA and plasma protein adducts in rats. Fractionation of the plasma proteins by Sephadex G-200 chromatography showed that all detectable bound aflatoxin was associated with a single peak corresponding to albumin (Wild et al. 1986)

The 60-year story of aflatoxin B1 (AFB) is a landmark for the field of toxicology, biomonitoring, chemoprevention, and public health interventions (Kensler et al. 2011; Wogan et al. 2012). Urinary metabolites, albumin adducts, DNA adducts, immunological effects, biochemical and biological mechanisms, and associations to disease such as liver cancer were studied over decades. The determination of DNA and albumin adducts (Fig. 3) was a key step in the evolution of this research (reviewed in (Sabbioni and Sepai 1998)). Animal experiments show that albumin adducts of AFB increase linearly with the dose, as do the DNA adducts in the liver (target organ) (Wild et al. 1986) (Fig. 4). For hemoglobin adducts, the studies with ethylene oxide (Ehrenberg et al. 1974) or with 4-aminobiphenyl (Green et al. 1984) are the landmarks for molecular epidemiology studies.

**Fig. 5:**
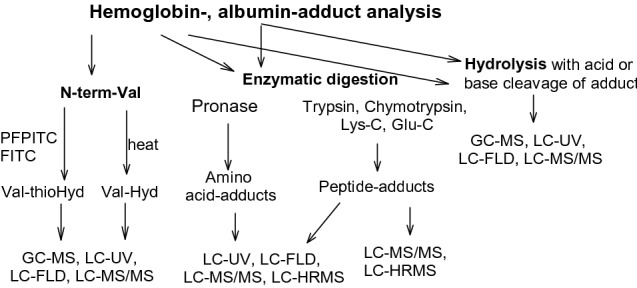
General approach for the analysis of hemoglobin (Hb) and albumin (Alb) adducts

Different approaches have been developed for the detection of albumin and hemoglobin adducts (Fig. 5). Before the year 2000, most methods were based on the cleavage of the adducts by base or acid. The hydrolyzed compound could then be determined by instruments available at that time. The analysis of peptide adducts was mostly performed using enzyme-linked immunosorbent assay (ELISA). As mass spectrometry developed, larger peptide adducts could be detected. In the past, the analyzed compounds were confirmed by synthetic standards. Now, researchers tend to (and at veracity’s peril) solely rely on the capabilities of mass spectrometry for the identification of compounds.

In the following, we present a short review of the progress made in regard to albumin and hemoglobin adduct determinations.

## Protein adducts

### Albumin adducts

#### In vitro* reactions of albumin*

For albumin, the N-terminus (aspartic acid) or different major amino acid side chains form adducts in vitro with reactive chemicals (reviewed in Goto et al. 2013; Rubino et al. 2009; Sabbioni and Turesky 2017; Tailor et al. 2016)) (Table 1). Albumin adducts of drugs (Tailor et al. 2016) (Table 1), organophosphorous compounds such as nerve agents (Golime et al. 2019) and pesticides were investigated. Especially, nerve agents were tested to discover long-term markers for nerve gas exposures (Golime et al. 2019). Drugs were tested in regard to potential adverse effects such as idiosyncratic effects (Baillie 2020; Stepan et al. 2011). In the field of environmental and occupational toxicology, albumin adducts were used as markers of exposure, of biologically effective dose for compounds causing oxidative damage, asthma, cancer, methemoglobinemia and other health effects.

**Table 1:** In vitro modification of human serum albumin (Alb) with chemicals and determination of the reacting amino acid by MS in tryptic digests

**D**^**ä,b,ß,f,p,n**^ AH**K**^**ä,b,ß,v,w**^ SE-6	VAH^n,q^ RF**K**^**ß,m,u,v,w,y**^ DLGE-16	ENFKALVLIA -26
FAQYLQQ**C**^**k,l,s,z,$**^ PF-36	EDHVK^w^ LVNEV-46	TEFAK^m,y^ TCVAD-56
ESAENCDKSL-66	**H**^**c,l,L,n,q**^ TLFGDK^ä,L^ LCT-76	VATLRETYGE-86
M^r^ ADCCAKQEP-96	ERNECFLQH^e,L^ K^y,L^-106	DDNPNLPR^p^ LV-116
RPEVDVM^r^ CTA-126	FH^L^ DNEETFLK^ß,m,t^-136	K^ß,m,y,ŷ^ **Y**^**c,d,e,ö,s**^ LY^c,ö^ EIARR**H**^**c,k,l,n,q**^-146
PY^d,ò,s^ FY^ò^ APELLF-156	FAK^g,m^ RY^o^ KAAFT-166	ECCQAADK^t,v,y^ AA-176
CLLPKLDELR^p^-186	DEG**K**^**ä,ß,j,m,u,v,w,x,y,ŷ**^ ASS^j^ A**K**^**f,h,j,l,m,y**^ Q-196	RL**K**^**a,ä,b,ß,c,e,f,g,h,I,j,m,n,u,v,w,y,ŷ,x**^ CASLQK^m^F-206
GERAF**K**^**ß,m,u,v,w**^ AWAV-216	AR^m,p^ LSQR^g^ FPK^u,v,w^A-226	EFAEVS^d,e^K^m,L^ LVT-236
DLTK^t^ VH^c,k,l,L^ TECC-246	H^c,L^ GDLLECADD-256	RADLAK^m^ YICE-266
NQDSISSK^ß^ LK^ß,m^-276	ECCE**K**^**ß,m,u,t**^ PLLEK^**m**^-286	S^d,e^ H^l^ CIAEVEND-296
EM^r^ PADLPSLA-306	ADFVES^g^ K^u,v^ DVC-316	K^u,v^ NYAEAKDVF-326
LGM^r^ FLYEYAR-336	R**H**^**c,k,n,q**^ PDYSVVLL-346	LRLA**K**^**ß,c,e,f,h,m,u,w,y**^ TYETT-356
LEKCCAAADP-366	HECYAKVFDE-376	F**K**^**ß,m,y,ŷ,L**^ PLVEEPQN-386
LIK^y^ QNCELFE-396	QLGEY^d^ K^m^ FQNA-406	LLVR^e,p^ Y^d,^^**o**^ TK^a,ä,b,ß,m^ **K**^**a,ä,b,ß,c,m,n,u,v,w**^ VP-416
QVSTPTLVEV-426	SR^m,p^ NLG**K**^**ä,ß,c,u,v,w,y,ŷ**^ VGSK^ä,ß^ -436	CCK^ß^ HPEAK^b,^^ß^^,m,v^ RM^r^-446
PCAEDY^d^ LSVV-456	LNQLCVLH^q^ EK-466	TPVS^g^ DRVTK^u^ C-476
CTES^e,g^ LVNRRP-486	CFS^g^ ALEVDET-496	Y^o^ VPKEFNAET-506
FTFH^l,L,q^ ADICTL-516	SEK^l,m^ ERQI **K**^**ä,b,ß,f,ŷ**^ **K**^**a,ä,b,ß ,c,e,l,m,n,t,u,v,w,y,ŷ**^ Q-526	TALVELVK^**ä,**ß^ HK^a,ä,ß,f^ -536
PKAT**K**^**ß,e,t,u,v,w,x,y,ŷ**^ EQLK^ß,m,L^ A-546	VM^**r**^ DDFAAFVE-556	K^ß,y^ CCK^ß^ ADDKET-566
CFAEEGK^b,ß^ K^b,ß^ LV -576	AASQAALGL-585	

The amino acid sequence of albumin is according to UniProtKB/Swiss‐Prot P02768 (ALBU_HUMAN), July 1, 2008, version 134. Molar ratios of compound to albumin are placed in parentheses:

^a^4,4’-Methylenediphenyl diisocyanate (MDI) (1:1) (Hettick and Siegel 2012), ^ä^MDI (10:1);

^b^2,4-toluene diisocyanate (24TDI) (1:1) (Hettick and Siegel 2011), 24TDI (10:1);

^c^tri‐*ortho*‐cresyl phosphate (CAS:78-30-8) (40:1) (Liyasova et al. 2012);

^d^10‐fluoroethoxyphosphinyl‐*N*‐biotinamidopentyldecanamide (FP‐biotin) (CAS:1811556-65-6) (1:1) (Ding et al. 2008);

^e^naproxen acyl coenzyme A thioester (CAS:475638-31-4) (2.2:1) (Olsen et al. 2003);

^f^“tolmetin glucuronide” (CAS:71595-19-2) (40:1) (Zia-Amirhosseini et al. 1995), as surrogate compounds the activated esters of tolmetin were prepared with 1-ethyl-3-(3-dimethylaminopropyl)-carbodiimide;

^g^benoxaprofen glucuronide (CAS:67472-42-8) (50:1) (Qiu et al. 1998);

^h^ “zomepirac glucuronide” (CAS:75871-31-7) (40:1) (Zia-Amirhosseini et al. 1995), as surrogate compounds the activated esters of zomepirac were prepared with 1-ethyl-3-(3-dimethylaminopropyl)-carbodiimide;

^i^acetylsalicylic acid (5.3:1) (Walker 1976);

^j^benzyl penicillin (CAS:61-33-6) (60:1) (Yvon et al. 1989, 1990);

^k^12-mesyloxy-nevirapine (CAS:1046462–02-5) as surrogate of the metabolite 12-sulfoxy-nevirapine (CAS:1046462-01-4) (5:1) (Meng et al. 2013);

^l^4‐hydroxy‐*trans*‐2‐nonenal (HNE) (5:1) (Aldini et al. 2006), ^L^(10:1) HNE (Campos-Pinto et al. 2019);

^m^glycation adducts (330:1) (Goto et al. 2013);

^n^1,2-epoxy-3,4-butanediol (1:10) (Lindh et al. 2005);

^o^nitration with peroxynitrite (110:1) (Goto et al. 2013); ö) Y138 or Y140, ò) Y148 or Y150;

^p^methylglyoxal (5:1) (Ahmed et al. 2005);

^q^CuSO_4_ + ascorbic acid (83:1) (Goto et al. 2013);

^r^H_2_O_2_ (167:1) (Goto et al. 2013);

^s^2-hydroxyamino-9*H*-pyrido[2,3-*b*]indole (HONH-AαC) (CAS:176853-90-0) (1:1) (Wang et al. 2015), sulfenamide, sulfinamide, and sulfonamide adduct formation occurred at Cys-34;

^t^malondialdehyde (100:1) (Ishii et al. 2008);

^u^tabun (CAS:77-81-6) (100:1) (Fu et al. 2020);

^v^ethyl-tabun (CAS:2351939-49-4) (100:1) (Fu et al. 2020);

^w^propyl-tabun (CAS: CAS:870124-37-1) (100:1) (Fu et al. 2020);

^x^amoxicillin (CAS:26787-78-0) (9:1) (Ariza et al. 2012);

^y^16α-hydroxyestrone (16αOHE1) (CAS:566-76-7) (1:1) (Charneira et al. 2020), ketoamine type adduct, ^ŷ^ketohydroxyamine type adduct;

^z^atrazine (6:1) (Chu and Letcher 2021);

^$^*N*-hydroxy-PhIP (CAS:124489-20-9) (1:1) (Peng and Turesky 2014)

In vitro modified albumin is digested with trypsin and analyzed by LC–MS/MS. The number of adducted amino acids increase with the amount of the chemical incubated with albumin. The molecular ratios used for most these experiments are far beyond the expected in vivo load of albumin. These experiments help to find eventual reactive hotspots on albumin. Sometimes, the intensity of the peaks is associated with a higher modification per mole of peptide, assuming that the detection response is the same for all molecules. The amino acids with most hits (≥ 4 different compounds, hot spots), obtained with the various compounds studied, are: Asp-1, Lys-4, Lys-12, Cys-34, His-67, Tyr-138, His-146, Lys-190, Lys-195, Lys-199, Lys-212, Lys-281, His-337, Lys-351, Lys-414, Lys-432, Lys-524, Lys-525, and Lys-541 (Table 1).

#### *Adducts formed with albumin *in vivo

**Fig. 6:**
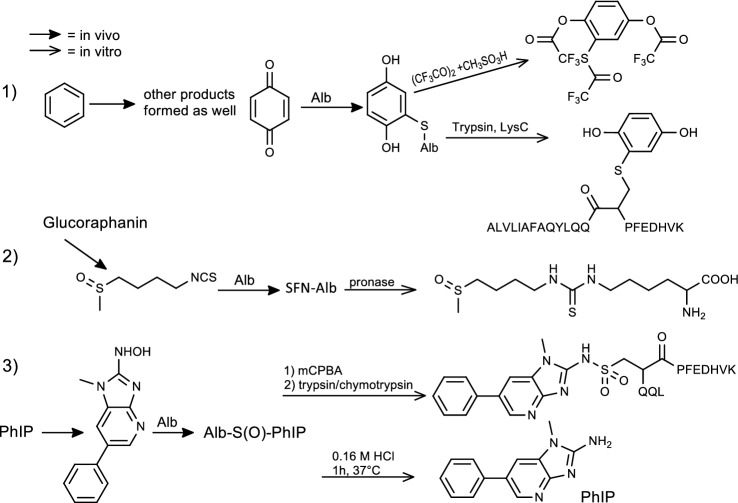
Typical analyses procedures for albumin adducts (Table 3). (**1**) Reactions and analysis of benzene adducts after chemical cleavage (Waidyanatha et al. 1998) or enzyme digestion (Smith et al. 2021); (**2**) Adduct of sulforaphane after eating broccoli (Kumar and Sabbioni 2010); (**3**) PhIP adducts found after oxidation with *meta*-chloroperoxybenzoic acid (mCPBA) and enzyme digestion (Peng and Turesky 2014) or acid hydrolysis (Bellamri et al. 2018)

For the analysis of in vivo samples, methods developed in the past used the technologies available at that time: ELISA, LC-UV, LC-FLD and GC–MS. Putative adducts were synthesized and then these adducts were searched in the in vivo samples. A very popular approach was the chemical cleavage of the adducts (Fig. 5, 6). Most adducts were cleaved by acid and/or base hydrolysis. The released chemical was extracted and analyzed for example by GC–MS (e.g., reviewed in arylamines (Sabbioni 2017)). With newer LC–MS/MS instruments, adduct analyses are performed with the detection of the intact adduct after enzymatic hydrolysis (Table. 2,3,1S). The aflatoxin B1 adduct with albumin has been part of many studies for 34 years (Groopman et al. 2008; Wogan et al. 2012). Here the typical evolution of methods took place: starting with ELISA tests, LC-UV, LC-FLD (reviewed in (Sabbioni and Sepai 1998) and LC–MS/MS (reviewed in (McCoy et al. 2008)). The sensitivity of the major albumin adduct AFB-Lys increases in the order of LC-UV, LC-FLD, ELISA and LC–MS/MS (McCoy et al. 2008). The compounds in Table 3 were ordered with ascending LOQ; it should be noted that many different definitions are used and applied for the terms LOD and LOQ (Shrivastava and Gupta 2011).

In albumin samples from humans and/or animals (Table 3, Table 1S, 2S, Fig. 6), the following compounds form an adduct with cysteine: a) environmental and occupational toxicants – benzene (Lindstrom et al. 1998; McDonald et al. 1993), pentachlorobenzene (Waidyanatha et al. 1994), styrene (Fustinoni et al. 1998), naphthalene (Waidyanatha and Rappaport 2008), acrolein (Witort et al. 2016); b) chemical warfare agents – sulfur mustard (Andacht et al. 2014), V-type nerve agents (Kranawetvogl et al. 2018); c) drugs – *N*-acetylaminophenol (Damsten et al. 2007); d) oxidative stress markers via cysteinylation (Regazzoni et al. 2013); e) natural products – pyrrolizidine alkaloids (Ma et al. 2019), estrogen quinones (Chen et al. 2020), aristolochic acid (Chan et al. 2021), the cooked meat carcinogen 2-amino-1-methyl-6-phenylimidazo[4,5-b]pyridine (PhIP) (Bellamri et al. 2018); f) pesticides – malathion (Yamagishi et al. 2021).

**Table 2:** Peptide adducts of cysteine-34 in albumin analyzed after digestion with different enzymes (Peng and Turesky 2014)

Enzyme	Peptide	LogD (pH)
Trypsin	ALVLIAFAQY LQQC*PFEDHVK	−10.41 (3.5–4.0)
Trypsin/chymotrypsin Chymotyrypsin	LQQC*PFEDHVK LQQC*PFEDHVK	−11.62 (3.5–4.0)
Chymotrypsin Trypsin/chymotrypsin	LQQC*PF LQQC*PF	−4.62 (5.0–6.5)
Proteinase K (40 °C) Proteinase K (55 °C)	QQC*PF QQC*PF	−5.33 (5.5–6.0)
Proteinase K (55 °C) Proteinase K (40 °C)	QC*PF QC*PF	−3.64 (5.0–6.0)
Aminopeptidase/prolidase	C*PF	−1.94 (5.5–6.0)
Aminopeptidase/prolidase	C*P	−3.06 (5.5–6.0)
Pronase E/leucine	C*	−2.79 (4.5–6.5)

Bold peptides were found to be the major peptides forming adducts for each proteolytic digestion system. ALVLIAFAQYLQQC*PFEDHVK (= T3 peptide) is obtained after digestion with trypsin (Li et al. 2011)

Pronase E is a mixture of endo- and exonucleases extracted from the extracellular fluid of *Streptomyces griseus*. LogD values were calculated with Marvin Sketch (Chemaxon) using the consensus method for logP value calculations

Adducts with lysines are found for: a) mycotoxins – AFB (Sabbioni 1990), aflatoxin G1 (Sabbioni and Wild 1991); b) isocyanates – 4,4’-methylenediphenyl diisocyanate (MDI) (Sabbioni et al. 2010), toluene diisocyanate (24TDI, 26TDI) (Sabbioni et al. 2012); c) isothiocyanates (ITC) derived from cruciferous vegetables – such as phenylethyl-ITC (PEITC) (Kumar and Sabbioni 2010), benzyl-ITC (BITC), allyl-ITC (AITC), and sulforaphane (SFN); d) environmental and occupational toxicants – tetrachloroethane (Pahler et al. 1999); e) oxidative stress markers – malondialdehyde (Colombo et al. 2016; Witort et al. 2016), formaldehyde (Regazzoni et al. 2017), f) endogenous compounds–glycation products (Altomare et al. 2021), and g) pesticides such as malathion (Yamagishi et al. 2021).

Adducts with tyrosines are found for: a) chemical warfare agents – sarin, soman, tabun, cyclosarin (Williams et al. 2007), tabun (Sun et al. 2017) and tabun-subtype nerve agents (Fu et al. 2019); b) pesticides – organophosphorus pesticides (von der Wellen et al. 2018), chlorpyrifos (Li et al. 2013), diazinon and dichlorvos (van der Schans et al. 2013); c) oxidative stress markers, e.g., yielding 3-nitrotyrosine (Delatour et al. 2002).

Adducts with histidines are found for: a) natural products - pyrrolizidine alkaloids (Ma et al. 2019), 1-methoxy-3-indolylmethyl glucosinolate (Barknowitz et al. 2014; Wiesner-Reinhold et al. 2019).

An adduct with tryptophan (Trp-215) was found in rodents given 4-aminobiphenyl (Skipper et al. 1985) or methleugenol (Nieschalke 2021), a natural compound of many plants.

In some in vivo studies, the levels of the same adduct type were compared between albumin and hemoglobin. In biological samples obtained after exposure to some xenobiotics, in general higher adduct levels were found in albumin than in hemoglobin: the cysteine adducts of naphthalene in mice (Waidyanatha and Rappaport 2008), the cysteine adducts of benzene in rats (Waidyanatha et al. 1998), the histidine adducts of 1‑methoxy-3-indolylmethyl isothiocyanate in mice (Barknowitz et al. 2014) (Fig. 6), the lysine adducts of isothiocyanates released from glucosinolates present in cruciferous vegetables (Kumar and Sabbioni 2010). The hydrolyzable adduct levels of arylamines are higher with hemoglobin than with albumin (Birner and Neumann 1988; Neumann et al. 1993). In contrast, for six radiolabeled arylamines tested in rodents, two had higher total adduct levels (hydrolyzable + non-hydrolyzable) with albumin than with hemoglobin.

In the newest studies, LC–MS/MS analyses after trypsin digestion is the method of choice to perform targeted and untargeted analyses (Grigoryan et al. 2016; Preston and Phillips 2019; Yano et al. 2020). However, it seems that applications are not going beyond small studies, since the detection levels of small molecules cannot be matched (Table 3). Therefore, for low level detection of chemicals more enzyme combinations were investigated to obtain shorter adducted peptides to increase the possibilities of separation of the adducted peptides from the unadducted peptides (Pathak et al. 2015). Thus, more facile enrichment and chromatographic separations of low molecular weight peptide adducts may be achieved than for the corresponding tryptic adducts, where the influence of the adduct on the logD is greatly diminished. In Table 2, the major peptides obtained with different enzymes is shown. The logD of the peptides was estimated by software.

**Table 3:** Albumin adducts found in vivo with a published limit of quantitation (LOQ) or limit of detection (LOD)

Compound [reactive intermediate]	Albumin adduct, work up	LogD (pH)	LOQ fmol/mg Alb	Method
Benzo[*a*]pyrene (BP) [BP-diol-epoxide]	Benzo[*a*]pyrene-r-7,t-8,t-9,c-10-tetrahydrotetrol^a^ derivatization with BSA/TCS	logD 1.44	0.01^a^	GC–MS
2-Amino-1-methyl-6-phenylimidazo[4,5-*b*]pyridine (PhIP) [NOH-PhIP]	Cys-(SO-PhIP), acid hydrolysis, PhIP	logD 2.09 (7.0)	0.05^ß^	LC–MS/MS
Aflatoxin B1 (AFB) [AFB-epoxide]	AFB-Lys^b^, pronase	logD −1.68 (4.0)	0.5 (LOD)^b^	LC–MS/MS
Benzene, [benzene-oxide]	S-phenyl-adduct / cleaved & derivatized with (CF_3_CO)_2_O + CH_3_SO_3_H	Phenyltrifluorothioacetate^c^	2 (LOD)^c^	GC–MS(NCI)
MDI (4,4 ‘-methylenediphenyl diisocyanate)	AcMDI-Lys^d^, pronase E MDI-Lys^e^, pronase E	logD 0.03 (3.0) logD 0.00 (6.0)	6.7^d^ 7.7^d^	LC–MS/MS
Benzene, [1,2-, and 1,4-benzoquinone (BQ)]	S-14BQ, S-12BQ adducts, cleaved & derivatized with (CF_3_CO)_2_O + CH_3_SO_3_H^f^		10 (LOD)^f^	GC–MS
26TDI (2,4-toluene diisocyanate)	3A2MP-Lys^g^, pronase E	logD −1.56 (6.5)	17^g^	LC–MS/MS
24TDI (2,4-toluene diisocyanate)	5A2MP-Lys^h^, 3A4MP-Lys^i^, pronase E	logD −1.56 (6.5)	17^g^	LC–MS/MS
[Phenylethylisothiocyanate (PEITC)]^x^	PEITC-Lys^j^, pronase E	logD −0.36 (4.5)	17.9^j^	LC–MS/MS
[Benzylisothiocyanate (BITC)]^y^	BITC-Lys^u^, pronase E	logD −0.65 (4.5)	18.8^j^	LC–MS/MS
[Sulforaphane (SFN)]^z^	SFN-Lys^k^, pronase E	logD −3.74 (4.5)	34.3^j^	LC–MS/MS
[3-(Isothiocyanatomethyl)-1-methoxy-1*H*-indole (MIM-ITC)]^&^	1-MIM-His^v^, pronase E	logD −1.87 (7.5)	67^v^	LC–MS/MS
[Allylisothiocyanate (AITC)]^§^	AITC-Lys^l^, pronase E	logD −1.64 (5.0)	113.7^j^	LC–MS/MS
[MIM-ITC]^&^	3-MIM-His^w^, pronase E	logD −1.53 (7.5)	280^v^	LC–MS/MS
PhIP [NOH-PhIP]	LQQC(-SO2-PhIP)PFEDHVK)^m^ Trypsin/chymotrypsin	logD −10.75 (3.5)	300^m^	LC–MS/MS
Sulfur mustard	[S-HETE]-CPF^n^, pronase E	logD −1.97 (5.5)	157^t^, 1000^t1^ 2000^t2^	LC–MS/MS
Nitrogen mustard	HN1-CPF^o^, pronase E HN2-CPF^p^, pronase E HN3-CPF^q^, pronase E	logD −2.70 (8.5) logD −3.07 (8.5) logD −3.76 (8.0)	5000^o^ 5000^o^ 1000^o^	LC–MS/MS
NEM (*N*-ethylmaleimide)^$^	ALVLIAFAQYLQQC(-NES)PFEDHVK^r^, trypsin	logD −10.40 (3.5)	30400^r1^	LC–MS/MS
1,4-Benzoquinone (14BQ)^$^	ALVLIAFAQYLQQC(-14BQ)PFEDHVK^s^ Trypsin + LysC	logD −10.25 (3.5)	43800^s^	LC-HRMS

The names and the structures of the adducts are in Table S2

^a^(Frank et al. 1998), CAS:61490-66-2; *N*,*O*-bis(trimethylsilyl)-acetamide (BSA) with 5% trimethylchlorosilane (TMCS); ß) (Bellamri et al. 2018)

^b^(McCoy et al. 2008), CAS:131919-04-5, structure in Fig. 3, name in Table S1;

^c^(Rynoe et al. 2003), no CAS number, [4-(2,2,2-trifluoroacetyl)oxy-3-(2,2,2-trifluoroacetyl)sulfanyl-phenyl] 2,2,2-trifluoroacetate;

^d^(Sabbioni et al. 2010), CAS:1200446-92-9;

^e^CAS:1200446-89-4;

^f^also 1,2-benzoquinone-adducts, (Waidyanatha et al. 1998);

^g^(Sabbioni et al. 2012), CAS:1416719-29-3;

^h^CAS:1416719-28-2;

^i^CAS:1416719-26-0;

^j^(Kumar and Sabbioni 2010), CAS:1211456-36-8;

^k^CAS:1211456-38-0;

^l^CAS:1609242-21-8;

^m^(Peng and Turesky 2014), no CAS number;

^n^(Noort et al. 2000), CAS:775312-71-5, *S*-[2-[(2-hydroxyethyl)thio]ethyl]-CPF;

^o^(Yeo et al. 2008), CAS: 1016983-35-9, *S*-[2-[ethyl(2-hydroxyethyl)amino]ethyl]-CPF;

^p^CAS:428508-48-9;

^q^CAS:1016983-38-2;

^r^NEM-modified albumin (NES-Alb; expected modification:, *S*-(1-ethyl-2,5-dioxo-3-pyrrolidinyl)-L-cysteine (Preston et al. 2017); r1) calculated from the modification level (0.2% = 2.4 pmol) of the synthetic standard NES-Alb in a total of 79 µg Alb. LOQ 2.4 pmol/79 µg Alb = 43.8 pmol/mg;

^s^(Smith et al. 2021), an on column LOQ of 7 fmol/160 ng was listed and this value was converted for 1 mg albumin, + lysC = lysine endopeptidase;

^t^(Liu et al. 2015), LOQ was calculated from the lowest reportable limit obtained from plasma incubated with 1ng sulfur mustard /mL plasma, 1 ml of plasma 40 mg of albumin were assumed; t1) (Andacht et al. 2014); t2) (John et al. 2016);

^u^CAS:1211456-34-6;

^v^(Barknowitz et al. 2014), CAS:1536466-52-0;

^w^CAS:1536466-53-1;

x to & from cruciferous vegetables: ^x^from gluconasturtin, ^y^glucotropaeolin, ^z^glucoraphanin, ^§^sinigrin, ^&^1-methoxy-3-indolylmethyl glucosinolate;

^$^ in vitro synthesized standards, that were just characterized by MS

**Fig. 7:**
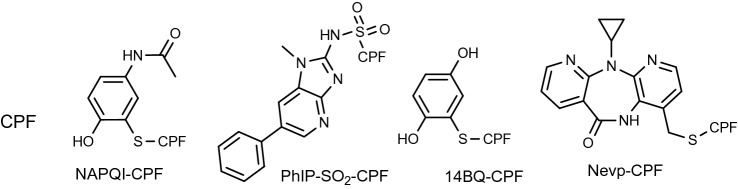
CPF adducts of 1,4-benzoquinone, *N*-Acetyl-*p*-benzoquinone imine (NAPQI) (Damsten et al. 2007), PhIP (Peng and Turesky 2014), and nevirapine (Antunes et al. 2010), yielding: NAPQI-CPF, 14BQ-CPF, PhIP-SO_2_-CPF, and Nevp-CPF. LogDs (pH 5.0–6.0) increase from left to right

Proteases, such as trypsin, can produce long peptides such as the T3-tryptic peptide A^21^LVLIAFAQYLQQCPFEDHVK^41^, whereas pronase digestion yields mono- di- or tripeptide Cys-containing adducts. The T3 peptide was used in most recent studies for a targeted and untargeted biomonitoring approach (Li et al. 2011; Preston et al. 2020). Combination of enzymes yields different lengths of peptides (Table 2). The logD values (pH dependent octanol–water partition coefficient) of long peptides not containing many hydrophobic amino acids are usually much smaller than the logD of smaller peptides such as CPF (Table 2). The logDs were predicted by software (www.chemaxon.com, Marvin Sketch 20.13, logP calculations in Chemaxon using the consensus mode). Such programs yield different results since for some structural features parameters are lacking. Hydrophobic adducts change the logD accordingly. The relative influence of hydrophobic adducts is larger in peptides with smaller logD values. For Cys adducts formed in longer peptides such as the T3 peptide, the logD values for 14BQ, NAPQI and nevirapine (Nevp) are -9.71, -9.25 and -8.41, respectively, (pH 4.0 = pH with the maximum level of the logD), compared to the unmodified T3 peptide with a logD of -10.4 at pH 4.0. The adducts formed of CPF (logD = -1.94) with NAPQI (NAPQI-CPF), 14BQ (14BQ-CPF) and nevirapine (Nevp-CPF) yield logD values of -1.03, -0.78 and + 0.07, respectively, at pH 5.5 (Fig. 7). Adducts of cysteine with NAPQI, 14BQ, and nevirapine yield a logD of -2.09, -1.63 and -0.78, respectively. In general, the logD of adducts which do not deprotonate or protonate in the the pH-ranges given in Table 2, increase with a constant amount in comparison to the unadducted peptides: for example for NAPQI, 14BQ and nevirapine with + 0.7, + 0.87, and + 2.01, respectively. Thus, more facile enrichment and chromatographic separations of adducts can be achieved with compounds with a higher logD. The highest logD were found for the tripeptide adducts of CPF. Other “lipophilic” hotspots (Table 1) could be for example FLK^195^K^196^YL (logD  = -1.13, pH= 9.5) or LK^199^CA (logD = -3.4, pH = 9.0), if they can be obtained in good yield by a combination of enzymes. The mass spectrometric properties of the shorter peptides are not dominated by the large number of amino acids present in the T3 peptide. However, smaller peptide fragments do not necessarily imply higher MS response (van den Broek et al. 2013; van den Broek et al. 2007). The effect of ionization suppression by co-eluting matrix components can be minimized by having the targeted adduct with a logD different than the bulk of the other components of the digest.

Probably, the LOQ for the albumin adduct of the adducted T3 peptide (ALVLIAFAQYLQQC(-14BQ)PFEDHVK) (Smith et al. 2021) with a logD of -9.25 could be lowered significantly using other enzyme combinations yielding 14BQ-CPF or 4BQ-C with a logD of -0.78 and -1.63, respectively, if the same digestion yields are obtained. To evaluate the digestion yields synthetic standards are needed. The same applies to the LQQC(-SO_2_-PhIP)PFEDHVK (Pathak et al. 2015). A combination of other enzymes would yield CPF and Cys adducts with logDs of -1.03 and -1.92, respectively.

In some cases, the decrease in sensitivity for analyses with adducts in the T3 peptide was further investigated. For the analysis of MDI-albumin adducts in workers, the single amino acid adduct (MDI-Lys) released after pronase digestion can be detected at lower levels (Sabbioni et al. 2010) than the MDI-peptide fragment released after trypsin digestion (Luna et al. 2014). In the case of the albumin adduct of sulfur mustard, the adducted T3 peptide ALVLIAFAQYLQQC(S-HETE)PFEDHVK could not be found in human samples (Noort et al. 1999), whereas the C(S-HETE)PF sulfur mustard adduct, obtained by pronase digestion, was identified in humans. The same applies to the adduct of PhIP with albumin. The peptide cannot be found in vivo but only after cleavage with acid (Bellamri et al. 2018; Wang et al. 2017). This is a consequence of the much lower LOQ for the cleaved product.

Thus far, the successes in measuring albumin-carcinogen adducts in humans have largely been with those adducts that are cleaved from albumin by acid or base treatment (i.e., Cys-BQ or BAP tetraols) (Rappaport et al. 2005; Sabbioni and Turesky 2017), or by the extensive digestion of albumin with a mixture of proteases to produce mono amino acid adducts (AFB-Lys adducts) (reviewed in Sabbioni and Turesky 2017)). The physico-chemical properties of these covalently adducted amino acids or carcinogen hydrolysis products are sufficiently distinct from non-modified amino acids or peptides such that selective enrichment procedures could be developed to isolate and assay the albumin adduction products. The employment of trypsin or other specific proteases to digest albumin produces defined peptides where sites of the toxicant adduction can be precisely located by MS/MS sequencing. These types of analyses provide valuable information about the sites of adduction to albumin, which are usually lost when digestions are done by pronase or acid/base hydrolysis, unless adducts are formed and retained to the solitary Cys-34 or Trp-214 residues of albumin.

An untargeted approach has been proposed by Rappaport et al. (Chung et al. 2014; Li et al. 2011) with the analysis of the tryptic digest containing Cys-34. The interpretation of massive MS-results remains difficult. Potential new adducts were not confirmed by synthetic standards. The experiments were all carried out with adducts that were not characterized by the standards of organic chemistry. The sensitivity of the method was not sufficient.

The logD values of different adducts used for in vivo analyses are listed in Table 3. At first sight, it appears that decreasing logD values are associated with an increasing LOQ. The response of the MS detectors depends also on the co-eluting matrix, the amount of fragmentation of the molecule, the proton affinity of the molecule, the chromatography and MS instrument parameters. In the case of negative ESI, the negative charge capture features of the analyzed molecule are important. This might explain the threefold difference of LOQ between AITC-Lys and SFN-Lys (Kumar and Sabbioni 2010).

### Hemoglobin adducts

#### In vitro* reactions with hemoglobin*

In comparison to albumin, not as many binding studies were performed with hemoglobin*.* Different specific reaction sites are known for hemoglobin (reviewed in (Rubino et al. 2009; Sabbioni 2017; Törnqvist et al. 2002)). In Table 4 and 3S, the results of the in vitro experiments are summarized. The data were obtained from tryptic digests of hemoglobin. Other enzyme combinations are possible for the analysis of hemoglobin adducts at β-Cys-93 (Table 5). As seen for albumin adducts, this might increase the sensitivity of the assay. However, the obtained fragments have very low logDs. In vitro reactions were performed with the following compounds: a) isocyanates – MDI, 24TDI; b) the reactive metabolites of occupational toxicants – styrene oxide, diepoxybutane; c) reactive metabolites of the drug – 12-mesyloxy-nevirapine as surrogate of the metabolite 12-sulfoxy-nevirapine, 16α-hydroxyestrone; d) oxidative stress markers – formaldehyde, glutathionylation, nitration, oxidation; d) skin sensitizers – 1-chloro-2,4-dinitrobenzene, 1,2-epoxy-3-phenoxypropane; e) chemical warfare agents – sulfur mustard; f) (heterocyclic) and aromatic amines – *N*-hydroxy-4-aminobiphenyl, *N*-hydroxy-aniline, 2-hydroxyamino-9*H*-pyrido[2,3-*b*]indole; g) oxidative stress markers. The main hot spots (≥ 4 hits of different compounds) are: α-Val-1, α-His-20, α-Tyr-24, α-His-45, α-Cys-104, β-Val-1, β-His-77, β-Cys-93, and β-Cys-112.

**Table 4:** Human hemoglobin (2α,2ß) chains taken from Uniprot (www.uniprot.org; α-chain P69905-1, ß-chain P68871-1)

**α-chain**		
^1^**V**^**a,ä,b,ß,c,ç,d,e,f,g,m,q,l,r,s**^ LSPADK^ä,r^ TNV	^11^K^ß^^,r^ AAWGK^ß,r^ VGA**H**^**c,ç,d,f,g,l,m,s**^	^21^AGEY^i^^,k,l,s^ GAEALE^d^
^31^RM^j^ FLSFPTTK^ä^	^41^TY^i^^,r,s^ FP**H**^**c,d,g,l,s**^ FDLSH^d,l,g^	^51^GS^r^ AQVKGHGK
^61^KVADALTNAV	^71^A**H**^**c,ç,d,f,g,l**^ VDDM^j^ PNAL	^81^SALS^l^ DLH^f,g^ AH^d,f,g^ K
^91^LRVDPVNFKL	^101^LSH^d*,u*^C^**c,ç**^^***,***^^d*,f,h,j,n,o^ LLVTLA	^111^AHLPAEFTPA
^121^VHASLDKFLA	^131^SVSTVLTSKY	R^141^
**β-chain**		
^**1**^**V**^**a,ä,b,ß,c,d,e,f,g,k,m,q,r,s**^ H^d,g^ LTPEEK^ä,r^ SA	^11^VTALW^j^ GK^ß,r^ VNV	^21^DE^g^ VGGE^g^ ALGR
^31^LLVVYPW^j^^,m^ TQR	^41^FFE^d^^,g^ SFGDLST	^51^PDAVM^j^ GNPK^r^ V
^61^K^ä^AHGK^ä^ K^a,ä^ VLGA	^71^FS^s^ DGLAH^c,d,g,l^ LDN	^81^LKGT^s^ FAT^s^ LS^l,m,s^ E
^91^LH^f^^,r,s^ **C**^**c,ç,d,f,h,k,j,n,o,p,s**^ DK^k^ L**H**^**c,ç,d,f,g,l**^ VDP	^101^ENFRLLGNVL	^111^V**C**^**c,ç,d,h,j,k,n,o,s**^ VLAH^l^ HFGK
^121^EFT^s^ PPVQAAY^i^	^131^QKVVAGVANA	^141^LAH^d^^,l,s^ K^ß,^^l,r^ YH^146^

Shown in bold are the hot spots for reaction products found with xenobiotics in vitro. Molar ratios of compound to hemoglobin are placed in brackets. a) MDI (1:1) (Mhike et al. 2013)

^ä^MDI (10:1) (Mhike et al. 2013)

^b^24TDI (1:1) (Mhike et al. 2016)

^ß^24TDI (10:1) (Mhike et al. 2016)

^c^styrene oxide (SO) (5:1) (Basile et al. 2002), α-His-45/His-50 and ß-Cys-93/His-97,respectively, are found alternatively alkylated, ç) SO (10^–4^:1) (Basile et al. 2002)

^d^diepoxybutane (10:1), ^*^Cys-104 or His-103 (Basile et al. 2002, 2001)

^e^formaldehyde (3:1 and 100:1) (Ospina et al. 2011)

^f^methylbromide (1:1) (Ferranti et al. 1996),

^g^sulfur mustard (50:1) (Hallez et al. 2021)

^h^GSSG (100:1) glutathionylation (Chen et al. 2014)

^i^peroxynitrite (150:1), nitration (Chen and Chen 2012; Kojima et al. 2016)

^j^H_2_O_2_ (1:1), oxidation (Kojima et al. 2016; Xiang et al. 2013)

^k^1-chloro-2,4-dinitrobenzene (5:1) (Ndreu et al. 2020)

^l^1,2-epoxy-3-phenoxypropane (5:1) (Ndreu et al. 2020)

^m^12-mesyloxy-nevirapine (100:1) (Antunes et al. 2010) a surrogate of the metabolite 12-sulfoxy-nevirapine, the valine adducts were analyzed by the Edman degradation procedure using phenylisothiocyanate

^n^*N*-hydroxy-aniline, (2:1) sulfinamide adduct, as in the whole blood experiments, only Cys sulfinamide modifications were observed for Hb in the presence of either GSH or a mixture of GSH and GSSG (Moller et al. 2017);

^o^*N*-hydroxy-4-aminobiphenyl CAS:6810-26-0 (3:1) (Pathak et al. 2016)

^p^2-hydroxyamino-9*H*-pyrido[2,3-b]indole (HONH-AαC) CAS:176853-90-0 (3:1) (Pathak et al. 2016)

^q^acetaldehyde (4300:1 to 430,000:1) (Birt et al. 1998)

^r^16α-hydroxyestrone (16αOHE1) (1:1), CAS:566-76-7, ketoamine type and ketohydroxyamine type adduct found, only ketoamine adduct at α-Ser-63 (Charneira et al. 2020)

^s^reaction with 4-methylene-2,5-cyclohexadien-1-one (5:1) yielding 4-hydroxybenzyl-adducts (Rajczewski et al. 2021), the valine adducts were analyzed separately (Degner et al. 2018)

**Table 5:** Peptide fragments containing Cys-93 (and/or Cys-123^a^) of the hemoglobin β-chain after hydrolysis with different enzymes (Pathak et al. 2016)

Enzyme	Peptide fragments	LogD (pH)
Trypsin	LLGNVLVC*^a^VLAHHFGK GTFATLSELHC*DKLHVDPENFR GTFATLSELHC*DK	−5.2 (9.0) −19.1 (3.5) −13.2 (3.5)
Lys-C	GTFATLSELHC*DK	−13.2 (3.5)
Glu-C	LHC*DKLHVDPE	−11.8 (3.5)
Chymotrypsin	HC*DKLHVDPENF ATLSELHC*DKL SELHC*DKL HC*DKL	−13.4 (3.5) −11.3 (4.0) −10.3 (4.0) −7.3 (7.0)

LogD values were calculated with Marvin Sketch (Chemaxon) using the consensus method for logP value calculations

#### Applications with the N-terminal valine adducts

**Fig. 8:**
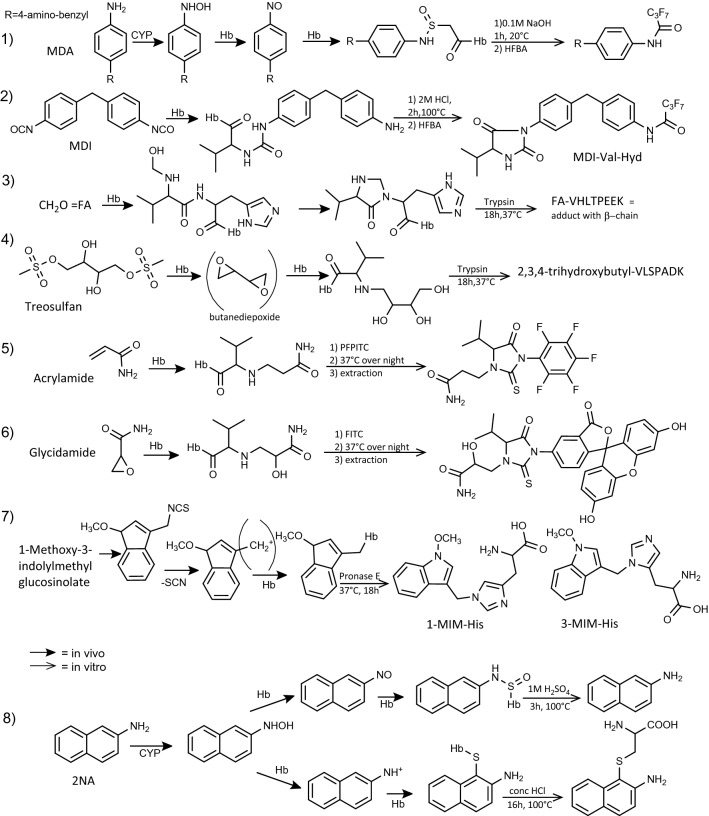
Typical analyses procedures for hemoglobin (Hb) adducts (Table 6): (**1**) Cys-93 adducts of 4,4’-methylenedianiline (MDA) released after base hydrolysis (Schutze et al. 1995). (**2**) 4,4,’-Methylenediphenyl diisocyanate (MDI) adducts with the N-terminal valine adduct released after acid hydrolysis (Gries and Leng 2013; Sabbioni et al. 2000). Such N-terminal valine adducts (Table 3S) have been found also for toluene diisocyanates (Sabbioni et al. 2001). (**3**) N-Terminal valine adduct of formaldehyde formed with the ß-chain of Hb and analyzed after trypsin digestion (Ospina et al. 2011; Yang et al. 2017). The adduct with the α-chain is not shown (FA-VLSPADK). Such imidazoline adducts have been determined for example also with acetaldehyde (Birt et al. 1998). (**4**) N-Terminal valine adducts of treosulfan analyzed after trypsin digestion (Boysen et al. 2019). The same adduct was formed with diepoxybutane (Kautiainen et al. 2000). (**5**) N-Terminal valine adduct analyzed using PFPITC for the modified Edman procedure and analyzed by GC–MS (Schettgen et al. 2016) or LC–MS/MS (Yang et al. 2018); (**6**) N-terminal valine adduct of glycidamide using FITC for the modified Edman procedure and analyzed by LC–MS/MS (von Stedingk et al. 2010). (**7**) Histidine adducts of 1-methoxy-3-indolylmethyl cation (Barknowitz et al. 2014). (**8**) Hb adducts of 2-naphthylamine resulting from 2-nitrosonaphthalene and the 2-naphthylnitreniumion intermediate (Linhart et al. 2021). The positive charge is delocalized over the molecule, and therefore as in this case, the electrophilic attack proceeded on a carbon

In human and animal studies (Fig. 8, Table 6, Table 3S), adducts with the N-terminal valine of hemoglobin (Carlsson et al. 2019, 2014) and with Cys-93 of the β-chain of hemoglobin (Pathak et al. 2016) were analyzed for example for alkylating agents (Törnqvist et al. 2002) and aromatic amines (reviewed in (Sabbioni 2017)), respectively. Hemoglobin was suggested for in vivo dose monitoring of alkylating agents as early as 1974 by Ehrenberg et al. (Ehrenberg et al. 1974; Osterman-Golkar et al. 1976). The method is based on the specific cleavage of adducts to N-terminal valines (alpha and beta chain) in hemoglobin (Törnqvist et al. 1986). For the GC–MS method, the globin is derivatized with pentafluorophenyl isothiocyanate (PFPITC) and after heating the adduct is cleaved from the rest of the protein. Several biomonitoring methods for the determination of N-terminal adducts of acrylamide, ethylene oxide, epichlorohydrin, glycidol, glycidamide, benzyl chloride, and others were validated in the German Working Group “Analyses in Biological Materials of the permanent Senate Commission for the Investigation of Health Hazards of Chemical Compounds in the Work” and the standard operation values are available online (https://onlinelibrary.wiley.com/doi/book/10.1002/3527600418) (Table 6). The procedures are presented in form of standard operating procedures and have been tested by other laboratories. The same derivatization with PFPITC was followed by LC–MS/MS analysis to determine the N-terminal valine adducts of acrylamide, glycidamide, and ethylene oxide (Yang et al. 2018) (Table 6, Fig. 8).

**Table 6:** Limit of quantitation (LOQ) for the determination of hemoglobin (Hb) adducts

Compound	Hemoglobin-adduct	LOQ, fmol/mg prot	Method	Instrument
4,4’-Methylenedianiline (MDA)	Base hydrolysis, MDA	0.06^a,v^	HFBA	GC–MS
Benzo[*a*]pyrene (BP)	BP-Hb, Benzo[*a*]pyrene-r-7,t-8,t-9,c-10-tetrahydrotetrol	0.3^b,w^ 250^a^^,u^	Acid^w^ hydr Base^u^ hydr	LC-FLD^w^ LC–MS/MS(APCI)^u^
4,4 ‘-Methylenediphenyl diisocyanate (MDI)	MDI-Val-Hyd^c^	0.15^b,c^	HFBA	GC-HRMS
Treosulfan [diepoxybutane]	2,3,4‐Trihydroxybutyl-VLSPADK^t^	0.4^b,t^	Trypsin	LC–MS/MS
Furfuryl alcohol	*N*-((Furan-2-yl)methyl)valine^d**^	0.9^a,d^	FITC	LC–MS/MS
Glycidol	*N*-(2,3-Dihydroxypropyl)valine^e^	0.7^a,e^	FITC	LC–MS/MS
Glycidol	*N*-(2,3-Dihydroxypropyl)valine^e^	25^b^^,e1,^^*^	PFPITC	GC–MS(NCI)
Glycidamide	*N*-(2-Hydroxy-2-carbonamide ethyl)valine^f**^	1^a^^,f^	FITC	LC–MS/MS
Glycidamide	*N*-(2-Hydroxy-2-carbonamide ethyl)valine^f**^	4.9^a,f1^(LOD), 6^b,f2,*^	PFPITC	LC–MS/MS^g^ GC-MS^f2^
Ethylenoxide	*N*-(2-Hydroxyethyl)valine^g**^	2^a^^,f^	FITC	LC–MS/MS
Ethylenoxide	*N*-(2-Hydroxyethyl)valine^g**^	6^b^^,f2,*^, 12.9^a,g^(LOD)	PFPITC	GC-MS^f2^, LC–MS/MS^g^
Acrylamide	*N*-(2-Carbonamide ethyl)valine^h**^	2^a^^,f^	FITC	LC–MS/MS
Acrylamide	*N*-(2-Carbonamide ethyl)valine^h**^	3.9^a,g^(LOD), 6^b,f2,*^	PFPITC^g,f2^	LC–MS/MS^g^, GC-MS^f2^
Acrylonitrile	*N*-(2-Cyanoethyl)valine^i**^	6^b^^,f2^^,^^*^	PFPITC	GC–MS
Sulfur mustard	N-(2-Hydroxyethylthioethyl)valine^s**^	7^b^^,s^ (LOD)	PFPITC	GC–MS
Ethyvinylketone	*N*-(3-Oxopentyl)valine^j^	15^a^^,j^	FITC	LC–MS/MS
Benzylchlorid	*N*-Benzylvaline^k**^	16^b,k,*^	PFPITC	GC–MS
Epichlorohydrin	*N*-(3-Chloro-2-hydroxypropyl)valine^l^	25^b,l,*^	PFPITC	GC–MS/MS(NCI)
1-Nitropyrene, 9-nitrophenanthrene, 1-nitronaphthalene	Hydrolysis, 1-aminopyrene, 9-aminophenanthrene, 1-aminonaphthalene	For all 25^a,u^ (LOD)		LC–MS/MS
Cyclophosphamide	*N*-[2-(2-Oxo-3-oxazolidinyl)ethyl]valine^m^	33^a^^,m^	FITC	LC–MS/MS
Formaldehyde	FA-VHLTPEEK^n^	670^a^^,n^&3400^a,n1^(LOD) 11300^a^^,n1^ (LOQ)	Trypsin	LC–MS/MS
Abacavir	Abacavir-Val^o^	700^b,o^	PITC	LC–MS/MS
*N,N*-Dimethylformamide, methylisocyanate	3-Methyl-5-isopropylhydantoin^p**^	1800^b^^,p,^^*^	Heat	GC–MS
*N,N*-Dimethylformamide, *N*-methylformamide	*N*-Methylcarbamoylvaline^r**^ *N*ɛ-(*N*-methylcarbamoyl)lysine^q**^	1000^b^^,r^ 5000^b^^,q,r^	Heat, acid^r^ Pronase^q^	LC–MS/MS

APCI=atmospheric pressure chemical ionization, FITC = fluorescein isothiocyanate, FLD=fluorescence detection, HBFA = heptafluorobutyric anhydride, HRMS=high resolution mass spectrometry, hydr = hydrolysis, NCI=negative chemical ionization, PFPITC = pentafluorophenyl isothiocyanate, PITC = phenylisothiocyanate

^a^Hb

^b^globin

^c^(Gries and Leng 2013) CAS:264285-90-7

^d^(Sachse et al. 2017) CAS:1531625-56-5

^e^(Hielscher et al. 2017) CAS:133278-70-3; e1) (Müller 2013)

^f^(von Stedingk et al. 2010) CAS:252663-74-4; f1) (Yang et al. 2018); f2) (Schettgen et al. 2016)

^g^(Yang et al. 2018) CAS:21768-51-4

^h^CAS:51078-53-6

^i^CAS:51078-49-0

^j^(Carlsson et al. 2015), only the FITC derivative has a CAS number

^k^(Lewalter et al. 2012) CAS:15363-84-5

^l^(Bader et al. 2013) CAS:223443-77-4

^m^(von Stedingk et al. 2014), *N*-[2-(2-oxo-3-oxazolidinyl)ethyl]-L-valine CAS:173962-82-8

^n^(CDC-NHANES 2020) N-terminal peptide (VHLTPEEK) of the ß-Hb-chain: N-(Hydroxymethyl)-VHLTPEEK (CAS:1307263-38-2), also the terminal amino acids of the α-Hb-chain was reported but not quantified in the CDC-NHANES studies, N-(hydroxymethyl)-VLSPADK, CAS:1307263-39-3; n1) (Yang et al. 2017)

^o^(Charneira et al. 2012) CAS:1350434-49-9

^p^(Käfferlein et al. 2016) CAS:74310-99-9

^q^CAS:848640-59-5

^r^(Mráz et al. 2006) CAS:84860-36-6

^s^(Xu et al. 2014) HETE-Val, CAS:190187-17-8

^t^(Boysen et al. 2019) CAS:2416700-26-8, main product

^u^(Wheelock et al. 2018)

^v^(Schutze et al. 1995)

^w^(Padros and Pelletier 2001);

^*^were validated in the German Working Group “Analyses in Biological Materials of the permanent Senate Commission for the Investigation of Health Hazards of Chemical Compounds in the Work” and the standard operation values are available online (https://onlinelibrary.wiley.com/doi/book/10.1002/3527600418)

^**^the compounds are commercially available, SciFinder search 23.7.21

The methods using PFPITC derivatization and GC–MS have been applied for the long-term health risks after accidental exposure using hemoglobin adducts of epichlorohydrin (Wollin et al. 2014), of acrylonitrile and ethylene in 2008 (Leng and Gries 2014). Another study using the method was performed to assess the exposure of acrylonitrile in the emergency responders of a major train accident in Belgium (Van Nieuwenhuyse et al. 2014). The validity of different biomonitoring parameters including the PFPITC derivatization was used for the assessment of occupational exposure to *N*,*N*-dimethylformamide (Seitz et al. 2018). In one event where Chinese male individuals were accidentally exposed to unknown chemicals, the N-terminal valine adduct of sulfur mustard was analyzed after PFPITC-derivatized N-terminal valine using GC–MS (Xu et al. 2014). A different approach was proposed by Mráz et al. (Mráz et al. 2018). The *N*-(2-hydroxyethyl)valine in globin of ethylene oxide-exposed workers was analyzed using total acidic hydrolysis and LC–MS/MS analysis.

Hemoglobin adducts of acrylamide and glycidamide have been determined in the large biomonitoring study by the CDC-NHANES. In the two sampling periods 2003–4 and 2005–6, 7101 and 7857 samples obtained from non-smokers were analyzed. The LOQ of acrylamide (glycidamide) adducts for the two sampling periods was 3 (4) and 0.11 (0.66) fmol/mg hemoglobin (CDC-NHANES 2021c). Additional samples from smokers were analyzed in 2013–2014 (CDC-NHANES 2019; CDC-NHANES 2021a). For acrylamide (n = 2348) and glycidamide (n = 2149) the LODs were 0.11 and 0.67 fmol/mg hemoglobin, respectively. In 2015/2016, acrylamide (glycidamide) was measured in 2413 (2267) samples (LOD = 0.11 (0.67)). The authors of the NHANES studies used PFPITC as derivatizing agent and analyzed the compounds by LC–MS/MS (Yang et al. 2018). The detailed standard operation procedures specified as laboratory methods are available for all biomonitoring NHANES studies (CDC-NHANES 2015–16; CDC-NHANES 2021b).

The classic Edman procedure using PFPITC was developed further in the laboratory of Törnqvist. For LC–MS/MS analyses, the derivatizing agent was changed to fluorescein isothiocyanate (FITC) (Rydberg et al. 2009; von Stedingk et al. 2010, 2011). Laboratories using this method should be aware of the structural changes of the FITC-derivatives depending from the pH (Rydberg et al. 2009). The adducts were synthesized and characterized with ^1^H-NMR, ^13^C-NMR and MS for the *N*-methylvaline (Rydberg et al. 2009), *N*-(3-oxopentyl)valine (Carlsson et al. 2015), *N*-benzylvaline, *N*-(2-hydroxybenzyl)valine, *N*-(3-hydroxybenzyl)valine, and *N*-(4-hydroxybenzyl)valine (Degner et al. 2018). Now, several adducts and internal standards can be purchased (Table 6, 3S). The new method with FITC was applied for the detection of N-terminal valine adducts with: glycidamide, ethylene oxide, and acrylamide (von Stedingk et al. 2010) (Table 6, 3S); glyoxal, methylglyoxal, acrylic acid, and 1-octen-3-one (Carlsson and Törnqvist 2016); 4-hydroxybenzaldehyde (Degner et al. 2018); ethyl vinyl ketone (Carlsson et al. 2015); and cyclophosphamide (Gernaat et al. 2021).

Monien et al. used the method to detect the N-terminal adducts of glycidol (Hielscher et al. 2017) (the same adduct forms for fatty acid esters of glycidol (Abraham et al. 2019)), furfuryl alcohol (Sachse et al. 2017), and estragole and anethole that yield the same estragole adduct (Bergau et al. 2021). The available LOQs for some of these compounds are listed in Table 6. The structures of these valine adducts are listed in Table 3S.

Analytical methods based on enzymatic digestion of hemoglobin and subsequent measurement of the resulting N-terminal peptide adduct by LC−MS/MS have been described for acetaldehyde (Birt et al. 1998), 1,2:3,4-diepoxybutane (Basile et al. 2002; Kautiainen et al. 2000), isoprene diepoxide (Fred et al. 2005), and formaldehyde (Ospina et al. 2011; Yang et al. 2017). The work of Birt et al. (Birt et al. 1998) is an exemplary of a chemical approach to discover the structure of a stable adduct with chemicals. In vitro experiments with acetaldehyde and the corresponding peptides of the N-terminal of α- and β-chains were performed and the structure of the imidazoline was characterized by NMR and MS (corresponding to the product for formaldehyde, see Fig. 8). These methods provide an alternative approach for the quantitative analysis of N-terminal adducts, especially for adducts not reacting with the Edman reagents. At the CDC, the same approach was applied to measure N-terminal adducts with formaldehyde. After trypsin digestion of the hemoglobin adduct, a peptide with the formaldehyde conjugated to the N-terminal-valine formaldehyde-VHLTPEEK was quantified (Table 6, Fig. 8), by LC–MS/MS (CDC-NHANES 2020; Ospina et al. 2011; Yang et al. 2017). Using this method, formaldehyde-hemoglobin adduct levels among the US population were determined in 2013–2014 in non-smokers (*n* = 2149) (CDC-NHANES 2021c) and smokers (CDC-NHANES 2021a) (*n* = 132). Applying a similar method, the adduct of treosulfan was used to detect the N-terminal adduct 2,3,4‐trihydroxybutyl-VLSPADK of the reactive intermediate diepoxybutane. After enzymatic digestion, the 7-mer adducted peptide was analyzed by LC–MS/MS (Boysen et al. 2019). The same approach was used to analyze N-terminal N-acylated and deaminated Val. Such modifications hinder the modified Edman procedure. The authors tried different enzymes - trypsin, chymotrypsin, endoproteinase Glu-C (V8), and AspN - to search for N-terminal peptides of the α-chain of hemoglobin. Asp-N gave short peptides and good digestion yields of VLSPADK and VLSPA. Adducted VLSPA was used as target molecule of choice (Usuzawa et al. 2021). The maximum logD of VHLTPEEK, VLSPADK, and VLSPA are -9.71, -7.73, and -3.62. Therefore, the high logD of VLSPA indicates the best peptide fragment for N-terminal adduct analyses.

The Törnqvist group used the FITC-method to perform targeted and untargeted analyses (Carlsson et al. 2019, 2014; Carlsson and Törnqvist 2016). The LOQs for the synthesized putative adducts found in humans are excellent (Table 6). The same research group proposed the untargeted analysis of adducts with the N-terminal valines of hemoglobin (Carlsson et al. 2014). The identification of new adducts is proceeding very slowly, since the untargeted screening by MS analyses generates enormous and complex datasets that are both difficult and time-consuming to interpret (Carlsson et al. 2017, 2019; Carlsson and Törnqvist 2016, 2017). In contrast to the other omics research topics such as proteomics and metabolomics, there is no commercial software to evaluate adductomics data: programs such as the SALSA algorithm (Badghisi and Liebler 2002) were used for a short time.

#### Applications with the cysteine adducts

Cysteine adducts of arylamines formed after exposure to the arylamines or the corresponding nitroarenes was reviewed recently (Sabbioni 2017). The reactive intermediates are nitrosoarene compounds that react with β-Cys-93 of hemoglobin. The resultant sulfinamide adducts can be hydrolyzed under mild conditions (0.1 M NaOH or 0.1 M HCl at room temperature) and the released arylamines can be detected at very low levels after derivatization with fluorinated acid anhydrides. In animals given radiolabeled arylamines (Neumann et al. 1993), the hydrolyzable part is related to the presence of a sulfinamide. 4-Chloroaniline, nitrobenzene, *N*-acetylaniline, benzidine, and 3,3’-dichlorobenzidine gave adducts that were hydrolyzable, in yields of 93%, 95%, 84%, 88%, and 32%, respectively, in animals sacrificed after 24 h (Neumann et al. 1993). Hemoglobin modified in vitro with radiolabeled 4-aminobiphenyl yielded only hydrolyzable adducts (Green et al. 1984). In vitro reactions with erythrocytes and *N*-hydroxyaniline confirmed the presence of only sulfinamides (Moller et al. 2017). However, unpublished work generated by Wolfgang Albrecht, a PhD student of Prof. Neumann, Department of Pharmacology and Toxicology, Würzburg, showed that the fraction of hydrolyzable hemoglobin adducts formed in rats decreased with time (Albrecht 1985). The hydrolyzable fraction compared to the totally bound radioactivity decreased from 1 day versus 7 days postdosing: for benzidine from 88.3 to 58.8%, for nitrobenzene from 98 to 52.3%, and for acetanilide from 58.3 to 39.2%. We postulate that the presumed sulfinamides may have undergone oxidation to form the chemically more stable sulfonamide in vivo (or via an in vitro/ex vivo experimental artifact). Arylsulfonamides (Mosher et al. 1958) are more stable than arylsulfinamides towards the hydrolysis conditions (0.1 M HCl at room temperature) used by Albrecht.

Chemical hydrolysis of hemoglobin adducts of xenobiotics with cysteine has been used for years for the detection of hemoglobin adducts of arylamines (reviewed in (Sabbioni 2017)). The LOQs of such an approach is lower than of peptide adducts. Hemoglobin β-Cys-93 sulfinamide and sulfonamide adducts of 4-aminobiphenyl were identified as peptide adducts in mice (Table 6, Fig. 8) by orbitrap MS following the proteolysis of hemoglobin with trypsin, Glu-C endoproteinase, or Lys-C endoproteinase (Pathak et al. 2016). The obtained β-Cys-93 containing peptides have very low logD values (Table 5). This hinders a separation of the adducts from the rest of the protein digest. This technique is not sufficiently sensitive and cleavage of the adduct by acid hydrolysis must be applied to detect the released 4-aminobiphenyl for human biomonitoring (Cai et al. 2017).

A new, sensitive method using LC–MS/MS was published for the analysis of hemoglobin adducts of polycyclic aromatic amines deriving from nitro-polyaromatic hydrocarbons present in polluted air (Wheelock et al. 2018). A novel method for source-specific hemoglobin adducts of nitro-polycyclic aromatic hydrocarbons was also described (Vimercati et al. 2020). Extensive comparisons were made to early biological effects (Vimercati et al. 2020).

Adducts in addition to cysteine sulfinamides were found in rats given 1- and 2-naphthylamine (NA) *S*-(1-amino-2-naphthyl)cysteine and *S*-(4-amino-1-naphthyl)cysteine were respectively found in rats given 1-NA and in those given 2-NA (Linhart et al. 2021) (Fig. 8). The novel aminonaphthylcysteine adducts were formed via naphthylnitrenium ions and/or their metabolic precursors in the biotransformation of naphthylamines. The positive charge is delocalized over the molecule, and therefore as in this case, the electrophilic attack proceeded on a carbon. The carcinogenic isomer 2-NA formed adducts at 100-fold-higher levels than the non-carcinogenic 1-NA isomer. These adducts are an additional new tool to monitor exposure to arylamines. These naphthylnitrenium adducts are present at a much higher level than the sulfinamide adducts formed through the nitrosoarene metabolite. The level of sulfinamide adducts in hemoglobin does not depend only from the formation of *N*-hydroxyarylamine (Sabbioni 1994) but also from the capacity to form the nitrosoarene in the erythrocytes according to the Kiese cycle (Kiese 1974). Therefore, for example, the mutagenic and/or carcinogenic potency of monocyclic arylamines correlate inversely to the levels of hemoglobin adducts (Sabbioni and Sepai 1995). In contrast, the hemoglobin adduct levels found in rats of bicyclic and bifunctional arylamines such as 4,4’-methylenedianiline, 4,4’-methylenebis(2-chloroaniline), 4,4’-oxydianiline, 4,4’-thiodianiline, 3,3’-dichlorobenzidine and benzidine correlate with the carcinogenic potency (Sabbioni and Schutze 1998). Roughly, the mutagenic and carcinogenic potency of arylamines is associated to the relative stability of the nitrenium ion, but not necessarily to the hydrolyzable hemoglobin (sulfinamide) adduct levels (Sabbioni and Sepai 1995; Sabbioni and Wild 1992) (Fig. 1S). The best correlations are found by including only similar compounds in the assessment, e.g., monocyclic arylamines as one category. More parameters have to be included for an overall prediction of the mutagenic and carcinogenic properties of arylamines (Benigni 2005; Benigni et al. 2007). In summary, the adducts of the nitrenium ions or the nitrosoarene originate from the same critical metabolite, the N-hydroxyarylamine. The nitrenium ion adducts are more stable and are more adaptable to the analysis of intact peptide adducts than the hydrolyzable sulfinamide adducts.

Compounds other than arylamines form adducts with cysteine of hemoglobin. For example, benzene (Rappaport et al. 2005), styrene (Fustinoni et al. 1998) and naphthalene (Waidyanatha and Rappaport 2008). Estrogen quinone-derived reaction products with cysteine, including 17β-estradiol-2,3-quinone and 17β-estradiol-3,4-quinone, were found at higher levels in hemoglobin cancer patients than in controls (Lin et al. 2014).

Adducts other than with cysteine or the N-terminal valine were found in mice given 1-methoxy-3-indolylmethyl glucosinolate (Fig. 7). Adducts with the metabolically released isothiocyanate were found with histidine (Barknowitz et al. 2014). Hemoglobin adducts of phenylethyl-ITC, benzyl-ITC, and sulforaphane with lysine were found in one subject eating cruciferous vegetables such as water cress, garden cress and broccoli (Kumar and Sabbioni 2010). Nitration, chlorination, and oxidation products were found in hemoglobin of breast cancer patients (Chen et al. 2021). Pyrrolizidine adducts with cysteine and histidine were found in humans (Ma et al. 2021).

In toxicological investigations, mostly adducts with the N-terminal valine or with cysteine in the β-chain were investigated. Hemoglobin adducts were analyzed after acid or base treatment, which yields the parent compound or a metabolite that can be extracted and separated from the biological matrix. This enables good sensitivities of the assays.

## Outlook

Many biomonitoring studies were performed using hemoglobin and albumin adducts in the last 40 years. Several compounds form adducts. With the progress of technology, researchers have wanted to take a global approach and have the vision to determine the individual exposome (Carlsson et al. 2019; Grigoryan et al. 2016). Methods are proposed to discover new chemicals on the adductome. The methods applied appear to be less sensitive than older methods (Table 3 and Table 6). Except for the large NHANES studies, most biomonitoring studies were performed with a small number of people. For analytical applications in forensic, food, drug, and clinical toxicology, accredited laboratories are performing the analyses with reference material. Therefore, in order for adduct research to progress, reference material should be used to make the analyses more reproducible. Several adducts are now commercially available. These are mostly adducts with single amino acids. To validate the analyses of adducts with larger peptides, the adducts should be synthesized and characterized, by at least ^13^C-NMR, ^1^H-NMR, UV, and MS. These synthetic peptide adducts along with the corresponding stable isotope labeled compounds should be used to evaluate the LOD and LOQs of the method. In addition, the sensitivity of the assay with larger peptides should be compared to the sensitivity of the assay with the classical assay after cleavage of the bond with the protein or after the digestion to the single amino acids. It might be worthwhile to compare the T3 peptide adduct analysis performance to the performance of the CPF adducts. Round robins should be organized to see if other laboratories measure comparable values. The detection limits of the synthetic compounds will show if the method is good enough to detect adducts in humans from environmental exposures.

Usually  < 1% (Sabbioni and Turesky 2017) of the dose of potential adduct-forming compounds bind with albumin in vivo. The estimated exposure levels (Wambaugh et al. 2013, 2014) should be taken from work performed at EPA (https://comptox.epa.gov/dashboard). Using these predicted exposures, a daily dose can be estimated. Assuming an adduction level of  < 1%, the daily albumin adduct level can be estimated from data obtained in animal experiments or from IVIVE predictions. If chronic exposure to the compound is likely and the adduct is stable, then the daily adduct level can be multiplied by 29. This yields the steady adduct level with albumin. If the detection limit of the assay performed with synthetic standards does not reach these levels, then it is highly unlikely to find adducts in environmentally exposed people.

To generate more preliminary data, the following road map is suggested. Instead of fishing in the dark, a more direct approach should be undertaken. Which compounds are important to include in biomonitoring studies? Databanks of potentially relevant compounds according to the lists published recently (Egeghy et al. 2016; Ring et al. 2019; Wang et al. 2020) should be used, a thorough prioritization of compounds should be undertaken, and the following values should be considered and introduced in the selection process: production volumes, toxicity, and predicted exposure levels (Blackburn et al. 2020; Dong et al. 2019; Sobus et al. 2019). From the selected list of compounds, the metabolism should be elucidated using experimental data, or predicted data from software such as QSAR Toolbox, Metaprint 2D, FAME, and Toxtree (Cronin et al. 2019; Kirchmair et al. 2015; Norinder et al. 2018; Shapiro et al. 2018; Suarez-Torres et al. 2020; Tan and Kirchmair 2014; Tian et al. 2018). In a next step, the structure of the potential adduct might be elucidated by the prediction of the reaction site by analogy and/or applying the concept of hard and soft nucleophiles, resp. electrophiles (LoPachin et al. 2012, 2019).

Practical skin sensitivity tests (OECD 2021a; OECD 2021b) are available. These are applied to reactions of chemicals to single amino acids or to small peptides with a free cysteine or lysine: a) the direct peptide reactivity assay, b) the amino acid derivative reactivity assay, and c) the kinetic direct peptide reactivity assay. The tests do not elucidate the structures of the reaction products, but only the disappearance of the original peptide after applying the chemical. Databanks of over 100 chemicals exist (Hoffmann et al. 2018; Urbisch et al. 2016, 2015). In addition, great efforts are put in prediction models for the assessment of new compounds especially for the cosmetic industry (Kimber 2021; Kleinstreuer et al. 2018; Natsch et al. 2020; Wareing et al. 2017). Synergies are possible between the researchers of laboratories interested in the development of methods to biomonitor people and to prevent release of skin sensitization products. Three compounds were tested recently to determine if skin sensitizing chemicals form albumin and hemoglobin adducts (Ndreu et al. 2020). It might be useful to introduce the short terminal peptides of the α- and ß-chain of hemoglobin, or the T3 peptide of albumin as probe for the reaction of potential sensitizing compounds.

For adduct analyses, presently the best sensitivity is reached with single amino acid adducts. Therefore, methods should be set up to aim at the amino acid hot spots discovered in vitro and confirmed partially in vivo. Some of these adducts are commercially available (Table 1S, 3S, 3, 6). Many compounds react with lysines. More compounds of significant potential environmental hazardous compounds should be added to the list of potential adduct-forming compounds. Starting with a diverse set of compounds, the targeted approach should be tested with pronase-digested albumin. Digestion of albumin to single amino acids yields for example lysine adducts (Kumar et al. 2009; Kumar and Sabbioni 2010; Sabbioni 1990; Sabbioni et al. 2012; Sabbioni and Wild 1991). This enables preliminary experiments to determine the sensitivity of the assay: LC–MS/MS, LC-HRMS and comparison to the predicted presence in the environment and the potential of adduct formation. In case of success, an untargeted approach might be tried to discover new compounds (e.g., lysine adducts) in samples collected from humans. The chemical properties of the potential adduct candidates should be predicted (logP, logD) with models to adjust the work up and conditions of the LC–MS/MS (preferably LC-HRMS) analyses. Untargeted MS analyses could be performed using the SAWTH-technique (Bruderer et al. 2018; Klont et al. 2020), neutral loss (LC–MS/MS (Barnaba et al. 2018; Dator et al. 2017)), and LC-HRMS (Carlsson et al. 2019). The newly discovered compounds identified by MS should be confirmed with synthetic standards. The same approach can be done with the other amino acid hot spots on hemoglobin and albumin.

Untargeted adductomics has not yielded new adducts that could be used in biomonitoring studies. The interpretation of the massive data appear to be too complicated (Carlsson et al. 2019). In addition, especially, for the analyses of albumin adducts, trypsin digestion yields large peptide fragments that cannot be analyzed with sufficient sensitivity (Preston et al. 2017). The method should be first tested with synthetic standards that have been characterized according to the standard protocols of organic chemistry.

Is untargeted adductomics feasible in the near future? The principle has potential as a tool to discover new markers of concern from both exposure and toxicological impact point of view. However, further improvements are necessary to make this approach fit-for-purpose with regard to human biomonitoring expectations, particularly for sensitivity (Hollender et al. 2017; Schymanski et al. 2015).

Alternative approaches to determine albumin and hemoglobin adducts are amino acid adducts of xenobiotics (valine, lysine) in urine (Mráz et al. 2020, 2016; Rabbani and Thornalley 2020), and mercapturic acids in urine (Bloch et al. 2019; Frigerio et al. 2019; Hanna and Anders 2020; Pluym et al. 2015; Wagner et al. 2006). However, like for most non-persistent chemicals, the urinary metabolites fluctuate substantially (LaKind et al. 2019; Pleil and Sobus 2013). For protein adducts, measurements have rarely been performed at different time points. Recently, Smith et al. (Smith et al. 2021) found a good intra class correlation coefficient (ICC = 0.91) for 14BQ adducts with albumin measured at 0, 56, and 84 days. For the other measured adducts without corresponding deuterated internal standard, the ICCs were below 0.62. However, for all products the adduct found in vivo was not confirmed and quantified with a synthetic standard. The low ICCs were justified with the varying air pollution measured as PM10, SO_2_ and NO_2_ concentrations during that period. The ICCs are used to show that the measurements give a reliable indication of the individual exposure. The following classifications are made for the reliability of the exposure measurements (LaKind et al. 2019): poor ICC < 0.4; fair to good ICC = 0.4 to < 0.75; and excellent for ICCs ≥ 0.75.

What are the future options of adductomics? The current tendency in molecular epidemiology is to collect data with the vision to be able to relate the exposome and other factors such as genetics and socioeconomic factors to disease (Vineis et al. 2020). However, the question arises as to how reliable and relevant the data are. Fishing into the data will lead to some potential relationships to one or more factors; however, how reproducible and significant are such exposure data? Working hypothesis should be built: what differences in adduct levels of a certain compound would lead to a disease? Perhaps using in vitro/in vivo relationships? Similar questions were raised and investigated in animal experiments. For example in aflatoxin research, it was of interest to determine the level of DNA adduct in the target organ relevant for liver tumor formation. Some relationship was found between species. The DNA chemical binding index of several chemicals was established in animals to evaluate a relationship between DNA binding level and likelihood of tumor formation (Lutz 1979; Otteneder and Lutz 1999). However, the vision of higher binding levels yielding more tumors could not be applied as a general model. In case–control studies with bladder cancer patients, significantly higher hemoglobin adduct levels were found (Skipper et al. 2003); however, the differences are so small that it is impossible to give a toxicological explanation. Originally, biomonitoring was developed to monitor workers. Most of the knowledge about exposure to chemicals in humans was discovered in workers. At the workplace, the occupational hygiene measures were improved, and the biomonitoring levels dropped for example in large German chemical companies such as Bayer with a great tradition in biomonitoring with scientists such as Miksche, Lewalter and now Leng. With compounds that are very toxic, such as aflatoxin, interventions were made, and the situation improved in many countries. Lead was reduced and the levels in children dropped. The levels diminished in the population.

How many chemicals of the > 400,000 are toxicologically relevant (Ring et al. 2019)? Is it possible to pick the dangerous candidates with biomonitoring studies, and if 1000 dangerous chemicals could be found, how significant are the health effects? And, if these chemicals are so dangerous, why were they not detected in the tests required to get them on the market? Would it not be easier to improve the OECD toxicological tests to avoid such compounds getting on the market? The chemicals on the market could be re-evaluated with new tests. In the outstanding EPIC studies (https://epic.iarc.fr/), a prospective study to link nutrition to cancer, numerous samples were collected, stored, and analyzed for many years. How clear and unambiguous are the results obtained from this study? What is more important—the poor nutrition, the socioeconomic factors, the environment, the lifestyle, the genes or just bad luck (Song et al. 2018; Tomasetti and Vogelstein 2015)?

Health data should be collected more thoroughly and included in geographical information systems. If some disease clusters are spotted, then it may be worthwhile to investigate more closely with biomonitoring studies. However, the difficulties of such approach might be hampered by the big ongoing globalization process. For example, often in Northern and Middle European countries, the hazardous work is performed by foreign workers. These workers go back to their home country and might get sick, and these cases are probably not recognized as occupational disease. In Switzerland, the cancer registries do not collect the information about the profession of the cases. Therefore, potential occupational links to the disease are missed.

Adductomic analyses are more work intensive and cost more than urinary analyses. Biomonitoring analyses cost at least 200 USD per sample and substance group (e.g., arylamines, http://www.ipasum.med.fau.de/files/2020/01/Preisliste.pdf) (Vorkamp and Knudsen 2019). Are the costs to monitor 100 classes of compounds and 100,000 people (= 2 × 10^7^ USD for one spot sample) helping to improve public health? Is it worthwhile to do one spot samples especially for urinary analyses that vary substantially? In summary, biomonitoring and adductomics should be used on a carefully selected small number of people that are monitored through the years as sentinels for exposure to xenobiotics. A more complete evaluation of exposure will be more effective using computer models, wastewater, water, air, and food analyses.

## Supplementary Information

Below is the link to the electronic supplementary material.

Supplementary file1 (DOCX 302 KB)
