# Supplementary material for: Quo vadis blood protein adductomics?
Source: Arch Toxicol. 2021 Nov 13;96(1):79–103. doi: 10.1007/s00204-021-03165-2 (PMC8748351; doi:10.1007/s00204-021-03165-2)
Supplement: Supplementary file 1 — Supplementary file1 (DOCX 302 KB) [file 204_2021_3165_MOESM1_ESM.docx]

**Supplemental Material**

**Quo vadis Blood Protein Adductomics?**

Gabriele Sabbioni^1,2*^, Billy W. Day^3,4^

^1^ Institute of Environmental and Occupational Toxicology, CH-6780 Airolo, Switzerland.

^2^ Walther-Straub-Institute of Pharmacology and Toxicology, Ludwig-Maximilians-Universität München,

D-80336 München, Germany.

^3^ Medantox LLC, Pittsburgh, PA 15241, USA.

^4^ ReNeuroGen LLC, Elm Grove, WI 53122, USA.

*Corresponding author

| **Table 1S. Albumin adducts** |  |
| --- | --- |
| **SFN-Lys** (CAS:1211456-38-0) *  *N*6-({[3-(Methylsulfinyl)propyl]amino}carbonothioyl)lysine  *N*6-[[[4-(Methylsulfinyl)butyl]amino]thioxomethyl]-L-lysine |  |
| **AITC-Lys** (CAS:1609242-21-8)*  *N*6-[(Allylamino)carbonothioyl]lysine,  *N*6-[(2-propen-1-ylamino)thioxomethyl]-L-lysine |  |
| **BITC-Lys** (CAS:1211456-34-6)*  *N*6-[(Benzylamino)carbonothioyl]lysine,  *N*6-[[(phenylmethyl)amino]thioxomethyl]-L-lysine |  |
| **PEITC-Lys** (CAS:1211456-36-8)*  *N*6-{[(2-Phenylethyl)amino]carbonothioyl}lysine  *N*6-[[(2-Phenylethyl)amino]thioxomethyl]-L-lysine |  |
| **3A4MP-Lys** (CAS:1416719-26-0)  *N*6-[[(3-amino-4-methylphenyl)amino]carbonyl]-L-lysine |  |
| **5A2MP-Lys** (CAS:1416719-28-2)  *N*6-[[(5-amino-2-methylphenyl)amino]carbonyl]-L-lysine |  |
| **3A2MP-Lys** (CAS:1416719-29-3)  *N*6-[[(3-Amino-2-methylphenyl)amino]carbonyl]-L-lysine |  |
| **MDI-Lys** (CAS:1200446-89-4)  *N*6-[[[4-[(4-Aminophenyl)methyl]phenyl]amino]carbonyl]-L-lysine |  |
| **AcMDI-Lys** (CAS:1200446-92-9)  *N*6-[[[4-[[4-Acetylamino)phenyl]methyl]phenyl]amino]carbonyl]- L-lysine |  |
| **16αOHE1-ketoamine-Lys** (CAS:792155-03-4)  *N6*-[(17b)-3-Hydroxy-16-oxoestra-1,3,5(10)-trien-17-yl]-L-lysine |  |
| **16αOHE1-hydroxyamine-Lys**  *N*-[(16a,17b)-3,16-Dihydroxyestra-1,3,5(10)-trien-17-yl]-L-lysine  (no CAS number) |  |
| **Sarin-Tyr** (CAS:1365420-03-6)*  *O*-[Methyl(1-methylethoxy)phosphinyl]-L-tyrosine |  |
| **Soman-Tyr** (CAS:1365420-06-9*  *O*-[Methyl(1,2,2-trimethylpropoxy)phosphinyl]-L-tyrosine |  |
| **VX-Tyr** (CAS:1365420-08-1)  *O*-(Ethoxymethylphosphinyl)-L-tyrosine |  |
| **HN1-CPF** (CAS:1016983-35-9)  *S*-[2-[Ethyl(2-hydroxyethyl)amino]ethyl]-L-cysteinyl-L-prolyl-L-phenylalanine |  |
| **HN2-CPF** (CAS:428508-48-9)  *S*-[2-[(2-Hydroxyethyl)methylamino]ethyl]-L-cysteinyl-L-prolyl-L-phenylalanine |  |
| **HN3-CPF (**CAS:1016983-38-2)  *S*-[2-[Bis(2-hydroxyethyl)amino]ethyl]-L-cysteinyl- L-prolyl- L-phenylalanine |  |
| **HETE-CPF** (CAS:775312-71-5)*  *S*-[2-[(2-Hydroxyethyl)thio]ethyl]-L-cysteinyl-L-prolyl-L-phenylalanine |  |
| **NAPQI-CPF** (CAS:949909-65-3)  *S*-[5-(Acetylamino)-2-hydroxyphenyl]-L-cysteinyl-L-prolyl-L- phenylalanine |  |
| **NAPQI1-CPF** (CAS:949909-64-2)  *S*-[2-(acetylamino)-5-hydroxyphenyl]-L-cysteinyl-L-prolyl-L-phenylalanine |  |
| Benzo[a]pyrene-r-7,t-8,t-9,c-10-tetrahydrotetrol  (7*R*,8*S*,9*R*,10*S*)-7,8,9,10-tetrahydrobenzo[*a*]pyrene-7,8,9,10-tetrol  (CAS:61490-66-2) |  |

*Commercially available compounds as listed in SciFinder (search 19.7.2021)

| **Table 2S. Compounds forming albumin adducts** |  |
| --- | --- |
| **Naproxen acyl coenzyme A thioester** (CAS:475638-31-4) |  |
| **Tolmetin glucuronide** (CAS:71595-19-2)* |  |
| **Benoxaprofen glucuronide** (CAS:67472-42-8)* |  |
| **Zomepirac glucunoride** (CAS:75871-31-7)* |  |
| **Amoxillin** (CAS:26787-78-0)* |  |
| **12‐Sulfoxyl‐nevirapine** (CAS:1046462-01-4) |  |
| **tri‐*ortho*‐Cresyl phosphate** (CAS:78-30-8)* |  |
| 10‐Fluoroethoxyphosphinyl‐*N*‐biotinamidopentyldecanamide (FP‐biotin, CAS:1811556-65-6) |  |
| **Tabun** (CAS:77-81-6)*  Phosphoramidocyanidic acid, *N*,*N*-dimethyl-, ethyl ester |  |
| **Propyl-Tabun** (CAS:870124-37-1)*  Phosphoramidocyanidic acid, bis(1-methylethyl)-, ethyl ester |  |
| **Ethyl-Tabun** (CAS:63815-60-1)*  Phosphonamidic acid, P-​cyano-​N,​N-​diethyl-​, ethyl ester |  |

| **Table 3S. Hemoglobin adducts** |  |
| --- | --- |
| **3A2MP-Val** (CAS:390824-61-0)*  *N*-[[(3-Amino-2-methylphenyl)amino]carbonyl]-L-valine |  |
| **5A2MP-Val** (CAS:390824-60-9)*  *N*-[[(5-Amino-2-methylphenyl)amino]carbonyl]-L-valine |  |
| **3A4MP-Val** (CAS:390824-59-6)*  *N*-[[(3-Amino-4-methylphenyl)amino]carbonyl]-L-valine |  |
| **3A2MP-Val-Hyd** (CAS:390824-73-4)  (5*S*)-3-(3-Amino-2-methylphenyl)-5-(1-methylethyl)-2,4-imidazolidinedione |  |
| **3A4MP-Val-Hyd** (CAS:223728-03-8)  (5*S*)-3-(3-Amino-4-methylphenyl)-5-(1-methylethyl)-2,4-imidazolidinedione |  |
| **5A2MP-Val-Hyd** (CAS:390824-72-3)  (5*S*)-3-(5-Amino-2-methylphenyl)-5-(1-methylethyl)-2,4-imidazolidinedione |  |
| **AcMDI-Val** (CAS:264285-86-1)  *N*-[[[4-[[4-(Acetylamino)phenyl]methyl]phenyl]amino]carbonyl]-L-valine |  |
| **MDI-Val** (CAS:1415322-26-7)*  *N*-[[[4-[(4-Aminophenyl)methyl]phenyl]amino]carbonyl]- L-valine |  |
| **MDI-Val-Hyd** (CAS:264285-90-7)  3-[4-[(4-Aminophenyl)methyl]phenyl]-5-(1-methylethyl)-2,4- imidazolidinedione |  |
| **N-(2-Furanylmethyl)-L-valine** (CAS:1531625-56-5)* |  |
| **Abacavir-Val** (CAS:1350434-49-9)  (5*S*)-1-[[(4*S*)-4-[2-Amino-6-(cyclopropylamino)-9*H*-purin-9-yl]-1-cyclopenten-1-yl]methyl]-5-(1-methylethyl)-3-phenyl-2-thioxo-4-Imidazolidinone |  |
| **16αOHE1-ketoamine-Val**  *N6*-[(17b)-3-Hydroxy-16-oxoestra-1,3,5(10)-trien-17-yl]-L-valine  (CAS-number of the valine ethyl ester 2481338-79-6) |  |
| **16αOHE1-hydroxyamine-Val**  *N*-[(16a,17b)-3,16-Dihydroxyestra-1,3,5(10)-trien-17-yl]-L-valine  (CAS-number of the valine ethyl ester 2481338-76-3) |  |

| **Table 3S. Hemoglobin adducts** |  |
| --- | --- |
| ***N*-(2,3-Dihydroxypropyl)-L-valine** (CAS:133278-70-3) |  |
| ***N*-(3-Chloro-2-hydroxypropyl)-L-valine**  (CAS:223443-77-4) |  |
| ***N*-(2-Carbonamideethyl)-L-valine** (CAS:51078-53-6)* |  |
| **HETE-Val, (**CAS:190187-17-8**)***  **N-[2-[(2-Hydroxyethyl)thio]ethyl]-L-valine** |  |
| ***N*-(2-Hydroxy-2-carbonamideethyl)-L-valine**  (CAS:252663-74-4)* |  |
| ***N*-(2-Hydroxyethyl)-L-valine** (CAS:21768-51-4)* |  |
| ***N*-(2-Hydroxypropyl)-L-valine** (CAS:91147-54-5)* |  |
| ***N*-(2-Cyanoethyl)-L-valine** (CAS:51078-49-0)* |  |
| ***N*-(Phenylmethyl)-L-valine** (CAS:15363-84-5)* |  |
| ***N*-[2-(2-Oxo-3-oxazolidinyl)ethyl]-L-valine**  (CAS :173962-82-8) |  |
| ***N*-[(Methylamino)carbonyl]-L-valine** (CAS:84860-36-6)* |  |
| ***N*-(Octyl-3-on)-L-valine** (CAS: unknown) |  |
| ***N*-(Pentyl-3-on)-L-valine** (CAS: unknown) |  |
| ***N*-[3-(4-Methoxyphenyl)-2-propen-1-yl]-L-valine** *N*-(isoestragole-3’-yl)-L-valine (CAS: unknown) |  |
| **N-[(4-Hydroxyphenyl)methyl]-L-valine**  (CAS:1531629-50-1)* |  |
| ***S*-Phenyl-L-cysteine** (CAS:34317-61-8)* |  |
| **Table 3S. Hemoglobin adducts** |  |
| ***N*ɛ-(*N*-Methylcarbamoyl)lysine** (CAS:848640-59-5)* |  |
| **3-MIM-His** (CAS:1536466-53-1)  3-[(1-Methoxy-1*H*-indol-3-yl)methyl]-L-histidine |  |
| **1-MIM-His** (CAS:1536466-52-0)  1-[(1-Methoxy-1*H*-indol-3-yl)methyl]-L-histidine |  |

*Commercially available compounds as listed in SciFinder (search 19.7.2021)

*Fig. 1S*. Correlation (r=-0.78, p<0.001) of the mutagenicity of the arylamine azides (n=19) compared to the relative stability of the arylamine-nitrenium ions (Sabbioni and Wild 1992). The experimental data were generated all from one laboratory. The nitrenium ion were obtained after photolysis of the azides. The stability of the nitrenium ions was calculated with the heat of formations (HF) of the nitrenium ions and the corresponding amines using the semiempirical program AM1. [Hf (arylamine-nitrenium) – Hf(arylamine)] – [Hf(aniline-nitrenium)-Hf (aniline)]. Correlations of the mutagenicity obtained (from different laboratories) with the arylamines (n=14, 5 missing compounds at the time of the publication=A, 26DMA, 245TMA, 246TMA, 1PYR) in Salmonella (with activation by rat liver S9) are only r=-0.48 (p=0.077), although correlation between the 2 mutagenicity measurements is excellent (r=0.94, p<0.001) for the 14 compounds. Abbreviations: A, aniline; 26DMA, 2,6-dimethylaniline; 245TMA, 2,4,5-trimethylaniline; 246TMA, 2,4,6,-trimethylaniline; 1NA, 1-napthylamine; 2NA, 2-naphthylamine; 4ABP, 4-aminobiphenyl; 2AF, 2-aminofluorene; 1PYR, 1-aminopyrene; 6CHRY, 6-aminochrysene; IQ, 2-amino-3-methylimidazo[4,5-*f*]quinoline; MelQ, 2-amino-3,4-dimethylimidazo(4,5-*f*)quinoline; IsoIQ, 2-amino-1-methylimidazo[4,5-*f*]quinoline;. MelQx, 2-amino-3,8-dimethylimidazo[4,5-*f*]quinoxaline; NI, 2-amino-3-methylnaphtho[1,2-*d*]imidazole; IsoNl, 2-amino-1-methylnaptho[1,2-*d*]imidazole; BI, 2-aminobenzimidazole; MeBI, 2-amino-1-methylbenzimidazole; PhIP, 2-amino-1-methyl-6-phenylimidazo[4,5-b]pyridine.

Sabbioni G, Wild D (1992) Quantitative structure-activity relationships of mutagenic aromatic and heteroaromatic azides and amines. Carcinogenesis 13(4):709-13
